# Awareness of Coronary Artery Disease Risk Factors Among Adults in the Jazan Region of Saudi Arabia

**DOI:** 10.3390/healthcare14162613

**Published:** 2026-08-19

**Authors:** Luai Alhazmi, Mohammed H. Ghasham, Mohammed A. Huzaymi, Ghada Ageeli, Fai Mahdi, Retaj A. Alameer, Raffan M. Hamzi, Reem Z. Alshalan, Raneem A. Bajobeir, Abdulbasit A. Jaabur, Ali W. Hakami, Omar Oraibi, Mohammed A. Madkhali, Mostafa Mohrag

**Affiliations:** 1 Department of Internal Medicine, College of Medicine, Jazan University, Jazan 45142, Saudi Arabia; 2 College of Medicine, Jazan University, Jazan 45142, Saudi Arabia; 3 Faculty of Medicine, University of Al-Jouf, Sakaka 72388, Saudi Arabia

**Keywords:** coronary artery disease, awareness, risk factors, behaviors

## Abstract

**Background**: Coronary artery disease (CAD) remains a leading global health burden largely driven by modifiable risk factors, and public awareness plays a critical role in its prevention and early risk reduction. However, gaps in knowledge and behavioral practice persist. **Objective**: This study aims to assess awareness of CAD risk factors and examine its relationship with the socio-demographic characteristics, lifestyle behaviors, personal medical history, and family history among residents of the Jazan Region, Saudi Arabia. **Methods**: A cross-sectional study was conducted among 559 participants between April and May of 2026. Data were collected using a structured questionnaire assessing awareness of behavioral, clinical, and non-modifiable risk factors. Awareness was scored out of 12 and categorized as poor (<50%), moderate (50–75%), or high (>75%). Chi-square tests were applied to analyze associations, and binary logistic regression analysis was performed to determine independent factors associated with high awareness. The results are expressed as adjusted odds ratios (aORs) with 95% confidence intervals. Statistical significance was set at *p* < 0.05, and data were analyzed using IBM SPSS Statistics v.29. **Results**: Among the 559 participants, 67.3% demonstrated high awareness, 29.9% moderate awareness, and 2.9% poor awareness of CAD risk factors. Awareness of modifiable factors was high, including smoking (96.8%), obesity (95.5%), hypertension (94.6%), and high cholesterol (93.4%), while non-modifiable factors such as family history (54.6%) and sex (60.3%) were less recognized. In the bivariate analysis, significant associations with awareness were found for age, body mass index (BMI), education, marital status, and employment status. Exercise was significantly associated with awareness (*p* = 0.020), with higher awareness levels observed in individuals with greater activity. High awareness was independently linked to a higher educational level (aOR = 1.76, *p* = 0.009) and a previous diagnosis of CAD (aOR = 6.28, *p* = 0.014). **Conclusions**: This study found moderate to high CAD risk factor knowledge among adult residents in the Jazan Region. Self-reported preventative practices differed among health behaviors, demonstrating that awareness alone may not always lead to healthy lifestyles. As the cross-sectional nature of the study precludes causal conclusions, more longitudinal, qualitative, or theory-informed research is needed to understand the drivers of healthy behavior adoption.

## 1. Introduction

Coronary artery disease (CAD) is one of the leading causes of morbidity and mortality (16%) worldwide, representing a major public health challenge [1]. CAD accounts for approximately 17.9 million deaths each year, and is a major contributor to early mortality across the world [2,3]. CAD remains a major global problem, particularly in low- and middle-income nations, driven by rapid urbanization, lifestyle changes, and an aging population. These factors increase the prevalence of risk factors such as hypertension, diabetes, obesity, and dyslipidemia, which are already high in places such as Saudi Arabia [4]. CAD is a health condition of serious concern in Saudi Arabia, characterized by a high incidence of modifiable risk factors. National data show that hypertension, diabetes mellitus, obesity, and dyslipidemia occur in about 25–30%, 18–25%, up to 39%, and 25–40% of adults, respectively, indicating that many people are at risk of heart disease [5,6,7].

Awareness of CAD risk factors in the general population is crucial to preventing and controlling the disease. Comprehensive understanding of these factors empowers individuals to embrace better lifestyles, pursue timely medical consultation, and comply with preventive strategies. Studies conducted in Saudi Arabia and other regions have reported varying levels of awareness about CAD and its risk factors among different segments of the general population [8]. For example, in Jeddah, Almalki et al. found that awareness varied considerably across different risk factors [9]; in the Al-Majma’ah region, 68.1% of participants showed good awareness, 28.4% fair awareness, and 3.5% poor awareness [10]; and a national study reported high awareness in 59.9% of respondents, associated with sex, age, education, and occupation [11]. However, data specific to the Jazan region remain limited. While awareness of common risk factors—including smoking, hypertension, and obesity—is very high, often exceeding 80–90% [12], awareness of other important factors—including physical inactivity, a sedentary lifestyle, poor dietary habits, and non-modifiable risk factors such as a family history of the disease and aging—tends to be lower, sometimes falling below 60% [13]. This difference highlights gaps in public knowledge, which may hinder effective prevention efforts.

Previous studies have revealed a knowledge–practice gap, with people still reporting engaging in risky behaviors such as a poor diet, lack of physical exercise, or overconsumption of sugary drinks despite being aware of their detrimental outcomes [14]. This suggests that knowledge alone does not always lead to effective behavioral modification, and additional interventions focused on social and environmental variables may be needed. There is currently a dearth of data on public awareness of CAD and its risk factors in the Jazan region. Hence, the current study aims to address the following question: What is the level of knowledge of the risk factors of CAD among the general population in the Jazan region, and what factors are connected with that awareness? The present study was designed to measure awareness of CAD risk factors and study its connections to socio-demographic variables, lifestyle factors, personal medical history, and family history of CAD in the general population in the Jazan region of Saudi Arabia. Assessing the level of knowledge and its drivers in this population can help in guiding region-specific health education initiatives, support early identification of risk factors, and inform focused preventive actions unique to the Jazan community. Despite the large number of research studies examining awareness of risk factors for cardiovascular disease, there is a lack of evidence from the Jazan region. Regional disparities in socio-demographic factors and access to healthcare services and health promotion programs may influence public awareness and preventive actions.

## 2. Materials and Methods

### 2.1. Study Design and Subject Selection

A cross-sectional descriptive study approach was used. The study was carried out between April and May of 2026 in the Jazan region, one of the 13 administrative regions of the Kingdom of Saudi Arabia, located in the southwest of the country along the Red Sea coast. According to the 2025 census, the region has about 5000 towns and villages and a population of about 1.4 million. The target population included all citizens of the Jazan region who were at least 18 years of age, regardless of sex. Medical physicians and medical students were excluded to avoid potential bias due to higher baseline knowledge. Individuals under the age of 18, those who refused to participate, or those who did not complete the questionnaire were also excluded.

### 2.2. Sample Size and Data Collection

The sample size was calculated using an online sample size calculator: https://www.calculator.net/sample-size-calculator.html (accessed on 1 April 2026), based on a population size of approximately 1.4 million adults. With a 95% confidence level, a 50% expected response distribution, a margin of error of ±5%, and an anticipated non-response rate of 5%, the minimum required sample size was determined to be 385 participants. A non-probability convenience sampling technique was utilized, whereby an electronic (online) questionnaire was distributed via social media platforms including WhatsApp, Telegram, and X, targeting individuals who met the inclusion criteria.

Data were collected using a self-administered questionnaire consisting of 33 items adapted from previously validated instruments [8]. Minor modifications were made to ensure cultural relevance, clarity of wording, and suitability for the local population in Jazan, based on expert input from local healthcare professionals. The final version underwent a pilot test with 30 participants, who were not included in the final analysis, in order to assess its clarity and reliability. After reviewing the modified tool, specialist and consultant cardiologists determined that each of the 12 items was relevant. The entire study sample (*n* = 559) was then used to assess the psychometric qualities of the 12-item awareness score. With a Cronbach’s alpha of 0.674 (Kuder–Richardson 20 = 0.674), as expected for dichotomously scored items, the internal consistency was deemed acceptable; corrected item–total correlations were positive for every item (range 0.14–0.45); and alpha-if-item-deleted values (0.630–0.675) verified that no single item diminished the overall reliability. Exploratory factor analysis was performed to assess the scale’s construct validity. With a substantial Bartlett’s test of sphericity (χ^2^ (66) = 870.0, *p* < 0.001) and a Kaiser–Meyer–Olkin measure of sampling adequacy of 0.794, the correlation matrix was confirmed to be appropriate for factoring. All 12 items loaded positively on the dominant first factor (eigenvalue 3.01), which accounted for 25.1% of the total variance (range 0.25–0.66; 11 of 12 at ≥0.30). Parallel analysis supported the factor’s retention, as its eigenvalue significantly exceeded the corresponding value derived from random data (3.01 versus 1.24). The questionnaire was structured into three sections. The first section included nine questions addressing socio-demographic characteristics such as age, sex, nationality, marital status, educational level, and employment status. The second section comprised 12 questions assessing general knowledge and awareness of CAD. The third section included 12 questions evaluating awareness of CAD risk factors, including modifiable and non-modifiable ones. Each CAD risk factor was assessed using a single structured questionnaire item asking participants whether they recognized the factor as increasing the risk of CAD. Lifestyle practices were assessed using self-reported questionnaire items, including smoking status, regular exercise, fast food consumption, and soft drink intake; it should be noted that these variables were used as a proxy measure of health-related behaviors and were not objectively verified. The collected data were entered into Microsoft Excel and stored securely, ensuring the complete anonymity of participants as no personal identifiers (e.g., names or identification numbers) were collected, thereby maintaining confidentiality and privacy.

### 2.3. Statistical Analysis

Statistical analysis was performed using IBM SPSS Statistics for Windows, version 29.0 (IBM Corp., Armonk, NY, USA). Descriptive statistics are used to summarize the data, with categorical variables presented as frequencies and percentages, while continuous variables are expressed as mean ± standard deviation. Awareness levels were assessed by assigning a score of one for each correct response and zero for incorrect responses, with a total possible score of 12. The total awareness score was converted into a percentage and categorized into three levels: poor (<50%), moderate (50–75%), and high (>75%). The 12 awareness items and the scoring method were adopted from Abukhudair et al. [8]; however, the percentage cut-off points were defined a priori by the research team using the scale midpoint and upper quartile, as the source study did not report specific numeric thresholds. Bivariate analysis using chi-square tests was conducted to examine associations between awareness levels and independent variables. Fisher’s exact test was applied where expected cell counts were below five. Age was categorized for the bivariate analysis and entered as a continuous variable in the regression model. Binary logistic regression analysis was performed to identify independent factors associated with high awareness, with the results reported as adjusted odds ratios (aORs) and 95% confidence intervals. The model was adjusted for age, sex, educational level, marital status, having children, family monthly income, previous diagnosis of CAD, and family history of CAD. For the regression analysis, awareness was dichotomized into high (>75%) versus moderate or poor (≤75%). Although awareness was measured on three ordered levels, ordinal logistic regression was not appropriate because the proportional odds assumption was not satisfied (likelihood-ratio test, χ^2^ = 16.59, df = 8, *p* = 0.035). Multinomial regression was also unsuitable, given the small number of participants in the poor-awareness category (*n* = 16). Multicollinearity was assessed using variance inflation factors; all values were below 5, indicating no significant collinearity. Model fit was evaluated using the Hosmer–Lemeshow test. A *p*-value less than 0.05 was considered statistically significant.

### 2.4. Ethical Consideration

Ethical approval for this study was obtained from the Scientific Research Ethical Committee (REC), Jazan University (HAPO-10-Z-001) on the 6 April 2026, with reference number 47/10/1844. Participation in the study was entirely voluntary, and electronic informed consent was obtained on the first page of the online questionnaire from all participants prior to data collection. All procedures were conducted in accordance with ethical standards to ensure the confidentiality, privacy, and rights of participants.

## 3. Results

### 3.1. Socio-Demographic Characteristics

The study included a total of 559 participants, with females constituting more than half (56.7%). The mean age was 37.9 ± 13.5 years. Regarding body mass index (BMI), 36.3% had normal weight and 32.6% were overweight, followed by obese (24.7%). Most participants had a bachelor’s degree (65.3%). The majority were married (64.4%), with 93.8% having children. In terms of employment status, 50.8% of participants were employed, 26.5% were unemployed, and 22.7% were students. Participants were almost equally distributed between village (49.9%) and city (50.1%) residence. Most were Saudi nationals (96.8%), and 33.3% reported monthly income exceeding 15,000 Saudi Riyals (SAR) (Table 1).

**Figure 1:**
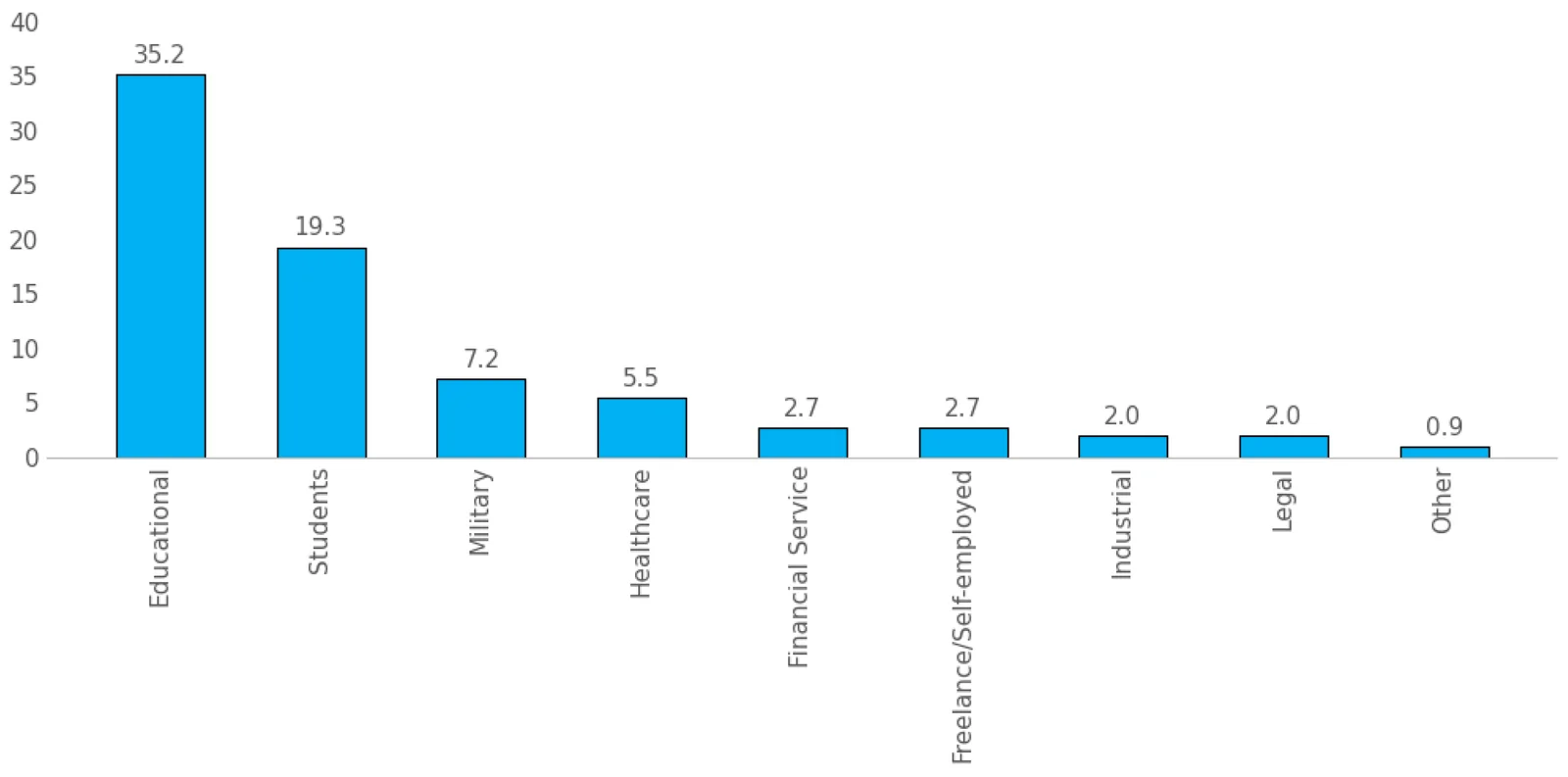
Distribution of employment sectors among the participants.

Figure 1 shows the distribution of participants across different work sectors. The educational sector constituted the largest proportion (35.2%), followed by students (19.3%), military (7.2%), and healthcare (5.5%). Employment in the industrial and legal sectors was low (2% each), while the “other” category accounted for the least (0.9%).

### 3.2. Awareness Level of CAD Risk Factors

Most participants demonstrated high awareness of key modifiable risk factors, including smoking (96.8%), fast food consumption (93.9%), obesity (95.5%), high cholesterol (93.4%), and high blood pressure (94.6%). However, awareness of non-modifiable risk factors was relatively lower, with 64.4% acknowledging age, 54.6% acknowledging family history, and 60.3% acknowledging male sex as risk factors (Table 2).

**Figure 2:**
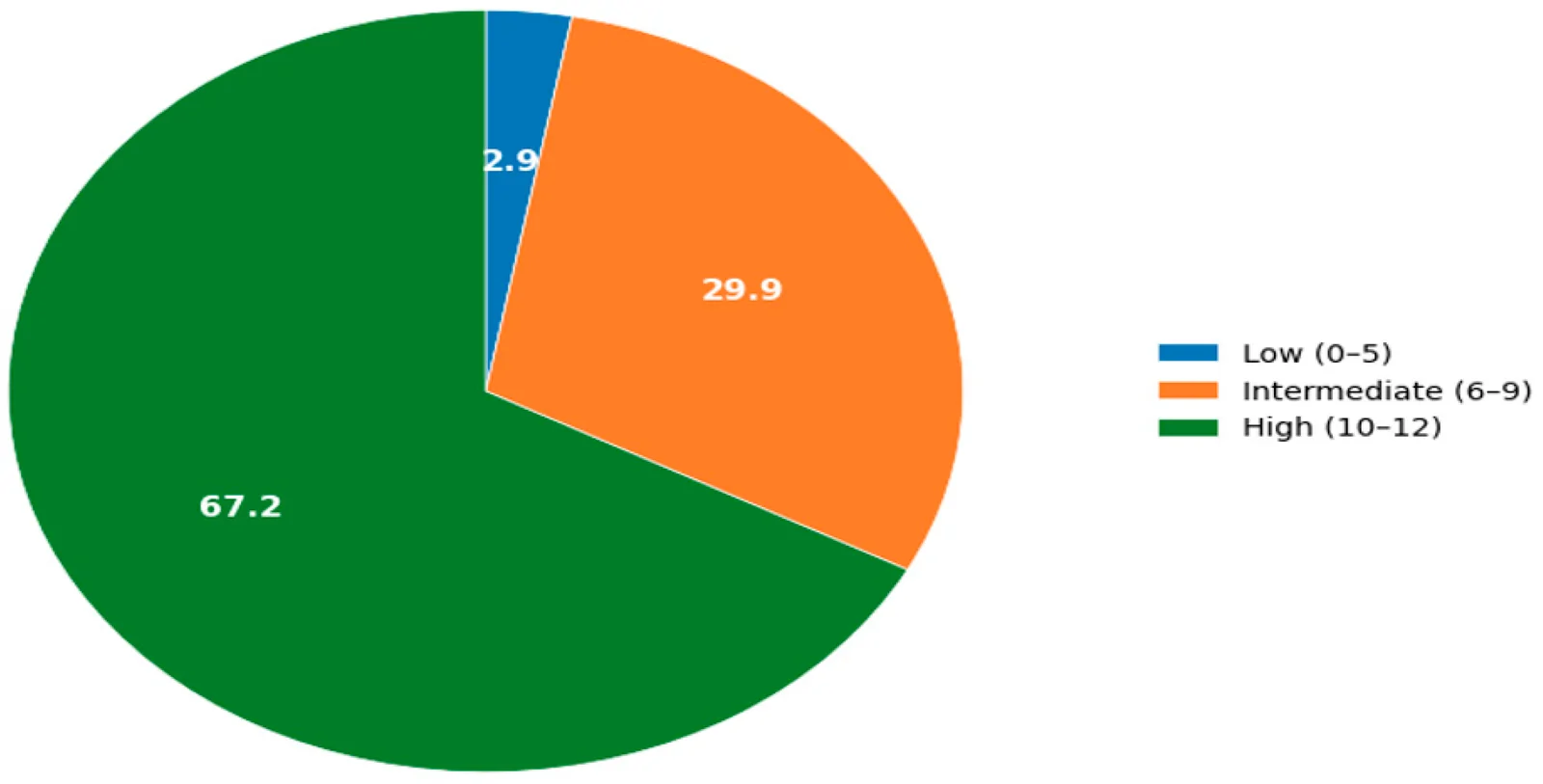
Awareness level about CAD risk factors among participants.

Figure 2 summarizes the overall awareness level of CAD risk factors among participants. Most participants demonstrated high awareness (67.3%), while 29.9% had a moderate level and 2.9% had a low level of awareness.

### 3.3. Distribution of Lifestyle Behaviors, Personal Medical Conditions, and Family History Among Study Participants

The majority of participants were non-smokers (88.2%). Only 35.8% engaged in regular exercise. A high proportion reported unhealthy dietary habits, with 61.9% consuming fast food and 61.7% drinking soft drinks. Furthermore, 4.7% had a diagnosis of CAD and family history was notable, with 169 (30.2%) reporting a CAD diagnosis among their relatives (Table 3).

### 3.4. Association Between Socio-Demographic Characteristics and Levels of Awareness

Table 4 summarizes the associations between socio-demographic characteristics and the levels of awareness of risk factors for CAD. Overall, most participants demonstrated moderate to high awareness across all categories. Age, BMI, educational level, marital status, and employment status were significantly associated with awareness. Higher awareness was more common among older participants, those with normal or higher BMI, those with a bachelor’s degree, married participants, and employed individuals (when compared with students). In contrast, sex, accommodation, nationality, and income showed no significant associations.

### 3.5. Association Between Awareness Levels and Lifestyle Behaviors

Table 5 summarizes the associations between awareness levels and lifestyle behaviors among participants. Exercise showed a significant association with awareness; in particular, individuals with higher engagement in regular exercise were more likely to have high awareness (73.0%), compared with those who did not exercise (64.1%). No other lifestyle behaviors showed statistically significant associations with awareness.

### 3.6. Logistic Regression Analysis

**Figure 3:**
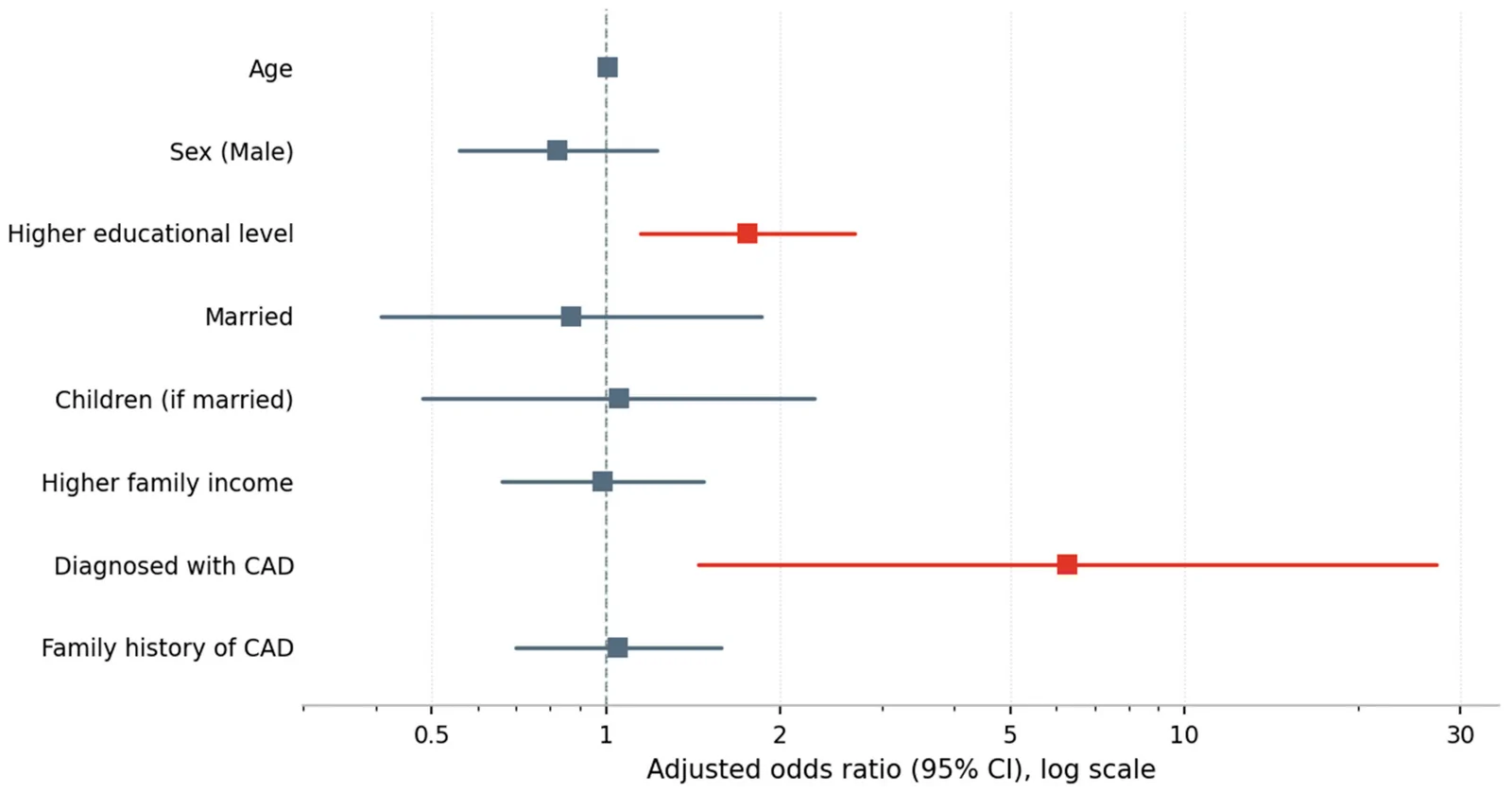
Adjusted odds ratios with 95% confidence intervals for factors associated with high awareness of CAD risk factors (*N* = 559). Squares indicate the adjusted odds ratio for each variable and horizontal lines the corresponding 95% confidence interval, plotted on a logarithmic scale. The vertical dashed line marks the null value (aOR = 1). Variables shown in red reached statistical significance (*p* < 0.05); those shown in grey did not. CI = confidence interval; aOR = adjusted odds ratio.

The results of the logistic regression analysis for factors associated with high awareness of CAD are displayed in Table 6. Higher educational level was independently associated with high awareness (aOR = 1.76, 95% CI: 1.15–2.69, *p* = 0.009). A previous CAD diagnosis was also associated with greater odds of high awareness (aOR = 6.28, 95% CI: 1.45–27.30, *p* = 0.014), though the wide confidence interval reflects the small number of diagnosed participants and warrants cautious interpretation. Age, sex, marital status, having children, income, and family history of CAD were not independently associated with awareness. The model showed acceptable fit (Hosmer–Lemeshow *p* = 0.30) and was statistically significant overall (*p* = 0.007). Figure 3 displays the adjusted odds ratios and their 95% confidence intervals for all variables entered into the model.

## 4. Discussion

CAD is one of the leading causes of morbidity and mortality worldwide, with modifiable behavioral and metabolic risk factors largely driving its burden [15,16]. Most of the study participants correctly identified smoking, obesity, hypertension, and high cholesterol as risk factors. This finding is consistent with previous studies such as that of Yang et al. (2010) [17], conducted among diagnosed CAD patients in South Korea, which indicated that these traditional risk factors are very well recognized due to extensive public health campaigns and physician counseling. Similarly, the awareness of physical inactivity, fast food consumption (93.9%), and soft drinks as risk factors was also high, which indicated improved public exposure to lifestyle-related health education [17,18]. In another study, Almalki et al. (2019) assessed the general population in Jeddah using a similar awareness questionnaire and showed that risk factors such as fast food, soft drinks, and family history were the most commonly identified, which were reported by 74.8%, 64.3%, and 47.2% of participants, respectively [9].

Despite this very high awareness, unhealthy behaviors were still very commonly reported, highlighting a critical gap between knowledge and practice, as reported widely in the previous literature. Ramachandran et al. (2016) showed that awareness alone is not sufficient to change behavior, as lifestyle choices are strongly influenced by environmental, habitual, and cultural factors [19].

The observed knowledge–behavior gap is probably multifactorial. While awareness of CAD risk factors was generally high, environmental factors, cultural dietary practices, limited opportunities for physical activity, socio-economic constraints and individual psychological factors (e.g., low perceived susceptibility and motivation) may impede healthier behavioral changes. These findings indicate that educational interventions are unlikely to produce significant reductions in cardiovascular risk without supporting behavioral and environmental strategies.

In contrast, awareness of non-modifiable risk factors—particularly family history and sex—was relatively low. This finding aligns with the study of Rasheed et al. (2019), conducted among hospital patients in Pakistan, which reported poor recognition of inherent risk factors and relatively poor knowledge about non-modifiable risk factors for CAD [20]. This discrepancy may be explained by the fact that public health messaging predominantly focuses on lifestyle changes while non-modifiable factors receive less emphasis, leading to reduced perceived susceptibility among individuals. This result is a very critical finding, demonstrating that a lack of awareness of inherent risks may reduce perceived susceptibility and delay preventive actions.

Regarding the overall awareness levels regarding CAD, most participants demonstrated high awareness (67.3%), while a minority (2.9%) had low awareness. This indicates that, although awareness is generally at an acceptable level, a substantial proportion of individuals still have insufficient knowledge, which requires targeted interventions. Similarly, in a national Saudi sample, Elsheikh et al. (2024) reported high awareness in 59.9%, medium awareness in 32.9%, and low awareness in 7.2%, comparable to our findings [11].

In the bivariate analysis, our results indicated various socio-demographic variables linked with awareness of CAD risk factors, such as age, BMI, education level, married status, and work status; in contrast, sex, accommodation, nationality, and income did not significantly influence awareness. Participants with normal or higher BMI had greater awareness than underweight participants, which may indicate increased health exposure or concern in the former groups. Similarly, those who had a bachelor’s degree had a higher level of awareness, consistent with past research in Saudi Arabia and other countries reporting that education increases access to health information and comprehension. Patients’ awareness levels showed connections with their educational qualifications (*p* = 0.036) but did not exhibit any association with BMI in the study of Elsheikh et al. (2024) [11].

The lack of associations with age and income contrasts with some previous studies reporting higher awareness among higher-income groups (Almutairi et al. 2024; general population in the Al-Majma’ah region) [10]. This difference may be due to widespread access to information through digital media, thus reducing traditional disparities, which has allowed individuals from various socio-demographic backgrounds to gain similar levels of awareness regardless of age or income. Overall, these findings suggest that awareness is becoming more evenly distributed across socio-demographic groups, though education remains an important independent association.

In the multivariable analysis, a previous CAD diagnosis and a higher educational level were found to be independently associated with high awareness. The link with education is consistent with Saudi and worldwide evidence that education increases access to and understanding of health information; for example, Elsheikh et al. (2024) [11] also reported education as a major correlate of awareness. The link with the previous diagnosis of CAD is clinically feasible, as those diagnosed with CAD will undergo direct counseling and have repeated interactions with health services; however, the small number of such participants limited the precision of this estimate. Although BMI and various socio-demographic characteristics were associated with awareness in the bivariate analysis, these associations became insignificant in the multivariable model, indicating that the correlations were dependent on educational level when all variables were considered together.

This study also revealed that, among the behavioral factors, only exercise was significantly associated with awareness of CAD risk factors. Participants with high awareness were more likely to engage in regular physical activity, suggesting that knowledge may positively influence certain health behaviors. This finding is consistent with previous studies reporting that health-conscious individuals are more likely to adopt active lifestyles. However, no significant association was observed with smoking, fast food, or soft drink consumption. This aligns with the existing literature, which highlights a persistent gap between knowledge and behavior, as individuals may remain engaged in unhealthy habits despite being aware of their risks [21]. This is likely due to environmental and cultural influences, such as peer pressure and the accessibility of healthier options. In their study, Ramachandran et al. (2016) demonstrated a positive association between awareness and healthy lifestyle behaviors [19]. However, the lack of associations with smoking and diet indicates that knowledge alone is insufficient to drive comprehensive lifestyle changes. It should be noted that behavioral practices were self-reported in this study and, therefore, should be interpreted with appropriate caution.

Another study recently conducted in the Jazan region evaluated the general public’s knowledge of cardiovascular disease risk factors and warning signs. While both that study and the present study focus on awareness of factors associated with cardiovascular health, they are completely unique in terms of their scope and design. The previous study was on general knowledge of cardiovascular disease and used a different questionnaire and sample, while the present study examines awareness of coronary artery disease in terms of specific risk factors, symptoms, prevention, and factors associated with it. Thus, the findings not only support previous studies but also add to the understanding of CAD-specific public awareness in the region [22].

### 4.1. Limitations

There are several limitations of this study. The cross-sectional design of this study limited the ability to establish causality between awareness and associated factors. The data obtained for this study were self-reported, which may introduce recall and social desirability biases, particularly for lifestyle behaviors. Therefore, the reported lifestyle behaviors may not fully reflect participants’ actual practices. The cut-off points used to classify awareness levels were pre-specified rather than derived from a validated threshold, which may limit direct comparison with studies applying different classifications. The awareness measure was not strictly unidimensional and had a modest level of internal consistency (α = 0.674). The study was conducted in a single geographic region, which may restrict extrapolation of the findings to other regions of Saudi Arabia. The use of non-probability convenience sampling through social media platforms may have introduced selection bias by preferentially recruiting younger, more educated, and technology-engaged individuals. Consequently, the study sample may not fully represent the general population of the Jazan region, limiting the external validity and generalizability of the findings. Finally, the study did not explore qualitative factors that influence behaviors, limiting understanding of the gap between awareness and practice.

### 4.2. Public Health Implications

This study’s findings support a multi-level approach to cardiovascular prevention. At the individual level, interventions should combine education with behavioral change counseling and motivational strategies. Awareness campaigns should be culturally tailored at the community level to highlight both modifiable and non-modifiable risk factors, and should encourage regular physical activity and healthy eating habits. At the policy level, efforts to improve access to healthy food choices, create opportunities for physical activity, and strengthen primary care-based cardiovascular risk screening may help to close the gap between awareness and healthy behavior.

## 5. Conclusions

This study revealed moderate to high awareness of CAD risk factors among adults in the Jazan region. However, self-reported preventive practices varied across different health behaviors, suggesting that awareness alone may not consistently be accompanied by favorable lifestyle practices. As the cross-sectional study design precludes causal conclusions, additional longitudinal, qualitative, or theory-informed research should be conducted to better understand the factors that influence the adoption of healthy behaviors.

## Figures and Tables

**Table 1:** Socio-demographic characteristics of study participants (*N* = 559).

		Frequency *N* (%)
Sex	Female	317 (56.7)
	Male	242 (43.3)
Age (Years)	Mean (SD)	37.9 (13.5)
	Range	18–76
BMI	Underweight	36 (6.4)
	Normal	203 (36.3)
	Overweight	182 (32.6)
	Obese	138 (24.7)
Educational Level	Secondary or less	151 (27.0)
	Bachelor’s	365 (65.3)
	Postgraduate	43 (7.7)
Marital Status	Single	171 (30.6)
	Married	360 (64.4)
	Widow/Divorced	28 (5.0)
Children (if married)	No	24 (6.2)
	Yes	363 (93.8)
Job Status	Unemployed	148 (26.5)
	Students	127 (22.7)
	Employed	284 (50.8)
Accommodation	Village	279 (49.9)
	City	280 (50.1)
Nationality	Non-Saudi	18 (3.2)
	Saudi	541 (96.8)
Family Monthly Income	<5000 SAR	116 (20.8)
	5000–9999 SAR	108 (19.3)
	10,000–15,000 SAR	149 (26.7)
	≥15,000 SAR	186 (33.3)

(*N*) Frequency; (%) Percentages; (SAR) Saudi Riyal; (BMI) Body Mass Index; (SD) Standard Deviation.

**Table 2:** Awareness of behavioral, clinical, and non-modifiable risk factors for CAD among participants (*N* = 559).

	No *N* (%)	Yes *N* (%)
Smoking	18 (3.2)	541 (96.8)
Lack of regular exercise	69 (12.3)	490 (87.7)
Eating fast food	34 (6.1)	525 (93.9)
Soft drinks	96 (17.2)	463 (82.8)
Age	199 (35.6)	360 (64.4)
Family history of heart disease	254 (45.4)	305 (54.6)
High cholesterol	37 (6.6)	522 (93.4)
Diabetes Mellitus	96 (17.2)	463 (82.8)
Obesity	25 (4.5)	534 (95.5)
Anxiety and stress	50 (8.9)	509 (91.1)
Male sex	222 (39.7)	337 (60.3)
High blood pressure	30 (5.4)	529 (94.6)

(*N*) Frequency; (%) Percentage.

**Table 3:** Distribution of lifestyle behaviors, personal medical conditions, and family history among study participants (*N* = 559).

	No *N* (%)	Yes *N* (%)
Are you a smoker	493 (88.2)	66 (11.8)
Do you exercise regularly	359 (64.2)	200 (35.8)
Do you eat fast food	213 (38.1)	346 (61.9)
Do you drink soft drinks	214 (38.3)	345 (61.7)
Diagnosed with CAD	533 (95.3)	26 (4.7)
Diagnosed with high cholesterol	433 (77.5)	126 (22.5)
Family history of high cholesterol	277 (49.6)	282 (50.4)
Diagnosed with diabetes	487 (87.1)	72 (12.9)
Family history of diabetes	190 (34.0)	369 (66.0)
Diagnosed with high blood pressure	463 (82.8)	96 (17.2)
Family history of high blood pressure	223 (39.9)	336 (60.1)
Family history of CAD	390 (69.8)	169 (30.2)

(*N*) Frequency, (%) Percentage.

**Table 4:** Associations between socio-demographic characteristics and awareness of CAD risk factors (*N* = 559).

		Low *N* (%)	Moderate *N* (%)	High *N* (%)	χ^2^	Sig. Value
Sex	Female	7 (2.2)	93 (29.3)	217 (68.5)	1.320	0.517
	Male	9 (3.7)	74 (30.6)	159 (65.7)		
Age	<25 Years	11 (7.9)	43 (30.9)	85 (61.2)	**19.090**	**0.004**
	25–34 Years	2 (2.0)	30 (30.0)	68 (68.0)		
	35–44 Years	1 (0.9)	38 (33.0)	76 (66.1)		
	≥45 Years	2 (1.0)	56 (27.3)	147 (71.7)		
BMI Category	Underweight	5 (13.5)	10 (27.0)	22 (59.5)	**18.885**	**0.004**
	Normal	3 (1.5)	67 (32.5)	136 (66.0)		
	Overweight	3 (1.6)	54 (29.5)	126 (68.9)		
	Obese	5 (3.8)	36 (27.1)	92 (69.2)		
Educational Level	Secondary or less	8 (5.3)	57 (37.7)	86 (57.0)	**13.109**	**0.011**
	Bachelor’s	6 (1.6)	99 (27.1)	260 (71.2)		
	Postgraduate	2 (4.7)	11 (25.6)	30 (69.8)		
Marital Status	Single	11 (6.4)	48 (28.1)	112 (65.5)	**13.564**	**0.009**
	Married	5 (1.4)	107 (29.7)	248 (68.9)		
	Widow/Divorced	0 (0.0)	12 (42.9)	16 (57.1)		
Children (Married)	No	0 (0.0)	14 (35.0)	26 (65.0)	1.007	0.604
	Yes	5 (1.4)	107 (29.5)	251 (69.1)		
Job Status	Unemployed	3 (2.0)	41 (27.7)	104 (70.3)	**11.275**	**0.024**
	Students	9 (7.1)	38 (29.9)	80 (63.0)		
	Employee	4 (1.4)	88 (31.0)	192 (67.6)		
Accommodation	Village	9 (3.2)	78 (28.0)	192 (68.8)	1.143	0.565
	City	7 (2.5)	89 (31.8)	184 (65.7)		
Nationality	Non-Saudi	0 (0.0)	7 (38.9)	11 (61.1)	1.143	0.565
	Saudi	16 (3.0)	160 (29.6)	365 (67.5)		
Family Income	<5000 SAR	6 (5.2)	31 (26.7)	79 (68.1)	6.369	0.383
	5000–9999 SAR	3 (2.8)	37 (34.3)	68 (63.0)		
	10,000–15,000 SAR	1 (0.7)	46 (30.9)	102 (68.5)		
	>15,000 SAR	6 (3.2)	53 (28.5)	127 (68.3)		

Significance values were derived using the chi-square test. Bold values indicate statistical significance at the *p* < 0.05 level.

**Table 5:** Associations between lifestyle behaviors and awareness of CAD risk factors (*N* = 559).

		Low *N* (%)	Moderate *N* (%)	High *N* (%)	χ^2^	Sig.
Smoking status	No	13 (2.6)	146 (29.6)	334 (67.7)	0.982	0.612
	Yes	3 (4.5)	21 (31.8)	42 (63.6)		
Exercise	No	8 (2.2)	121 (33.7)	230 (64.1)	**7.859**	**0.020**
	Yes	8 (4.0)	46 (23.0)	146 (73.0)		
Fast food consumption	No	4 (1.9)	56 (26.3)	153 (71.8)	3.712	0.156
	Yes	12 (3.5)	111 (32.1)	223 (64.5)		
Soft drink consumption	No	3 (1.4)	56 (26.2)	155 (72.4)	5.554	0.062
	Yes	13 (3.8)	111 (32.2)	221 (64.1)		

Significance values were derived using the chi-square test. Bold values indicate statistical significance at the *p* < 0.05 level.

**Table 6:** Logistic regression analysis results for factors associated with high awareness of CAD risk factors (*N* = 559).

	B	S.E.	Sig.	aOR	95% CI
Age	0.008	0.011	0.443	1.008	0.987–1.029
Sex (Male)	−0.193	0.201	0.338	0.825	0.556–1.224
**Higher Educational**	**0.564**	**0.217**	**0.009**	**1.758**	**1.149–2.689**
Married (Yes)	−0.139	0.387	0.720	0.871	0.408–1.860
Children (if married)	0.050	0.398	0.900	1.051	0.482–2.295
Higher Family income	−0.014	0.205	0.947	0.986	0.660–1.474
**Diagnosed with CAD**	**1.838**	**0.750**	**0.014**	**6.281**	**1.445–27.299**
Family history of CAD	0.049	0.208	0.813	1.050	0.699–1.579
Constant	0.092	0.291	0.752	1.096	—

Model fit: McFadden pseudo-R^2^ = 0.030; Hosmer–Lemeshow χ^2^ = 9.56, *p* = 0.297; overall model *p* = 0.007. Bold values indicate statistical significance at *p* < 0.05. All variance inflation factors < 5, indicating no multicollinearity.

## Data Availability

The original contributions presented in this study are included in the article. Further inquiries can be directed to the corresponding author.
